# Supplementary material for: Exploring causality between bone mineral density and frailty: A bidirectional Mendelian randomization study
Source: PLoS One. 2024 Jan 25;19(1):e0296867. doi: 10.1371/journal.pone.0296867 (PMC10810463; doi:10.1371/journal.pone.0296867)
Supplement: S1 File — (DOCX) [file pone.0296867.s001.docx]

**Supporting information**

Exploring causality between bone mineral density and frailty: a bidirectional Mendelian randomization study

**Supplementary Table**

**Supplementary Table** The detailed data information of the MR study for the causal relationship between BMD and FI.

| Type | Study/Consortium | Population | Sample size | Sample control | Datasets ID in the GWAS |
| --- | --- | --- | --- | --- | --- |
|  |  |  |  |  |  |
| e-BMD | MRC-IEU | European | 40,613 | NA | ukb-b-11364 |
|  |  |  |  |  |  |
| FA-BMD | GeFOS | European | 8,143 | NA | ieu-a-977 |
|  |  |  |  |  |  |
| FN-BMD | GeFOS | European | 32,735 | NA | ieu-a-980 |
|  |  |  |  |  |  |
| LS-BMD | GeFOS | European | 28,489 | NA | ieu-a-982 |
|  |  |  |  |  |  |
| FI | UKB/ TwinGene Sweden | European | 164,610 / 10,616 | NA | ebi-a-GCST90020053 |

Abbreviations: MR, Mendelian randomization; BMD: bone mineral density; FI: frailty index; e: heel; FA: forearm; FN: femoral neck; LS: lumbar spine; UKB: UK Biobank; GeFOS: Genetic Factors in Osteoporosis Consortium; MRC: Medical Research Council; IEU: Integrative Epidemiology Unit.

**Supplementary Figures**


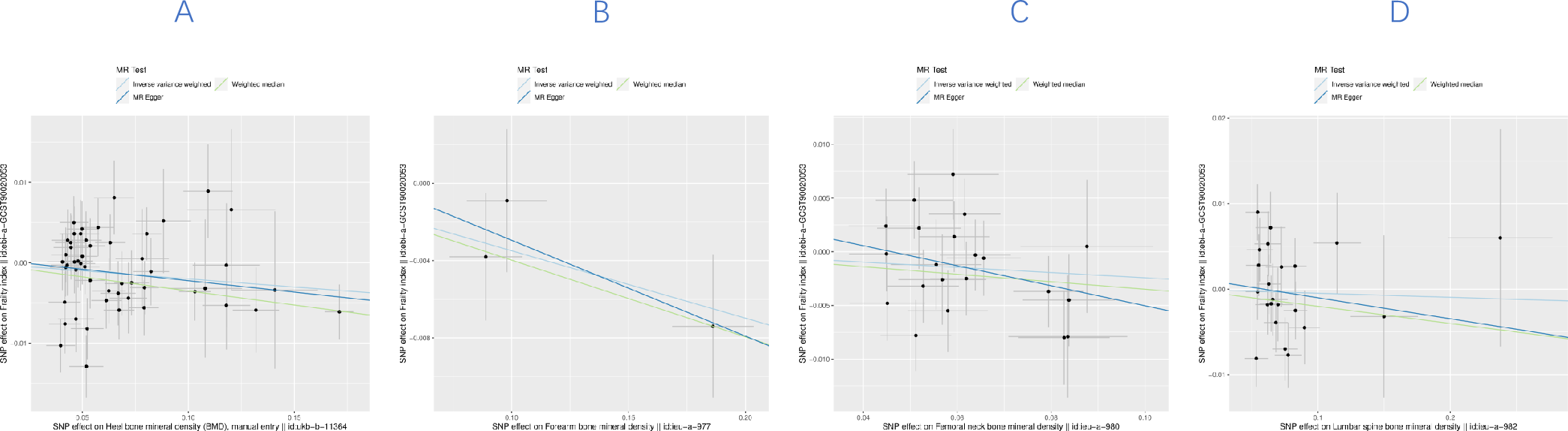


**Supplementary Figure 4.** Scatter plot for the effect of bone mineral density (BMD) on the frailty index (FI). (A) e-BMD, (B) FA-BMD, (C) FN-BMD, (D) LS-BMD

FN: femoral neck; LS: lumbar spine; FA: forearm; e: heel.


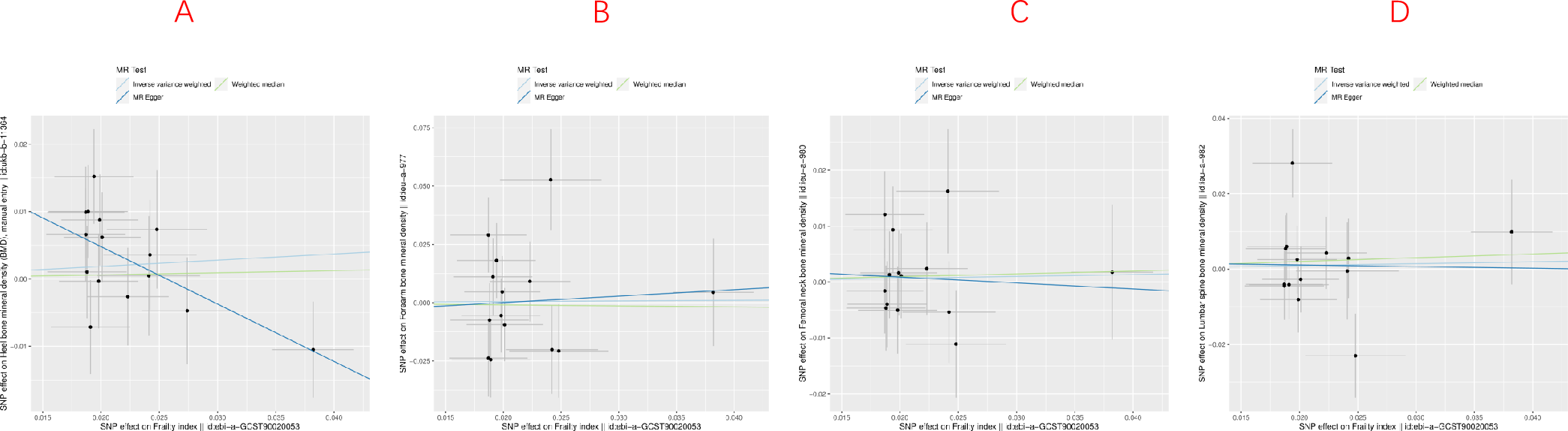


**Supplementary Figure 5.** Scatter plot for the effect of FI on the BMD. (A) e-BMD, (B) FA-BMD, (C) FN-BMD, (D) LS-BMD


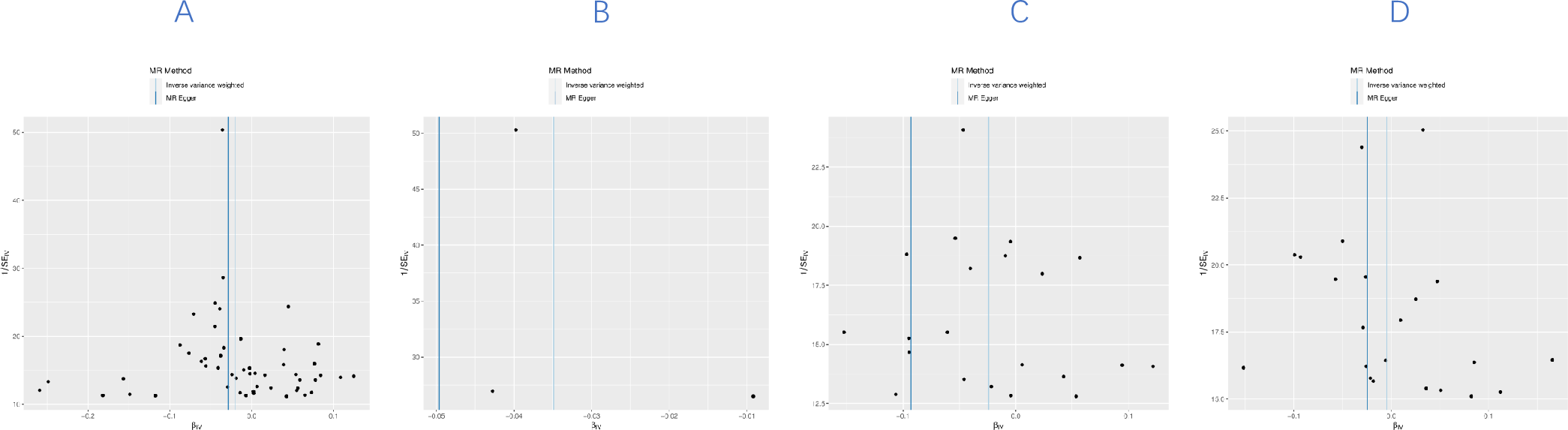


**Supplementary Figure 6.** Funnel plot for the effect of BMD on the FI. (A) e-BMD, (B) FA-BMD, (C) FN-BMD, (D) LS-BMD


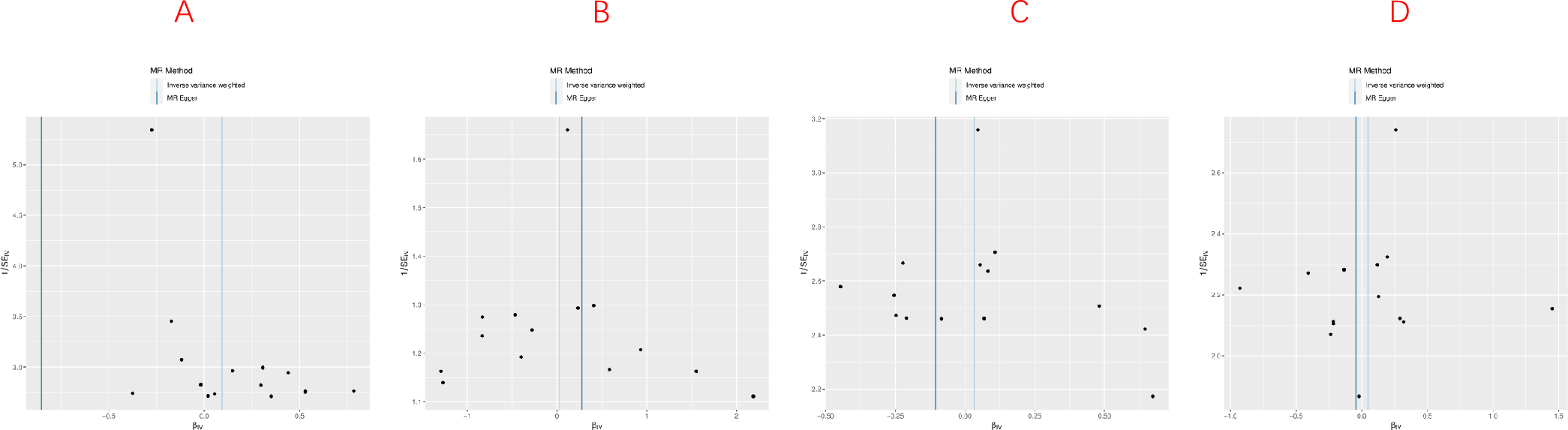


**Supplementary Figure 7.** Funnel plot for the effect of FI on the BMD. (A) e-BMD, (B) FA-BMD, (C) FN-BMD, (D) LS-BMD

R Code

#Take e-BMD (exposure) and FI (outcome) as an example:

install.packages("devtools")

devtools::install_github("MRCIEU/TwoSampleMR")

library(TwoSampleMR)

library(MRPRESSO)

library(ggplot2)

ao <- available_outcomes()

exposure_dat <- extract_instruments(c(' ukb-b-11364'))

outcome_dat <- extract_outcome_data(exposure_dat$SNP, c('ebi-a-GCST90020053'))

dat <- harmonise_data(exposure_dat, outcome_dat, action = 2)

res <- mr(dat,method_list = c("mr_ivw","mr_weighted_median","mr_egger_regression"))

generate_odds_ratios(res)

write.csv(res, file="res.csv")

run_mr_presso(dat)

mr_heterogeneity(dat)

mr_pleiotropy_test(dat)

res_single <- mr_singlesnp(dat)

p1 <- mr_scatter_plot(res, dat)

p1[[1]]

ggsave(p1[[1]], file="p1.pdf", width=7, height=7)

p2 <- mr_forest_plot(res_single)

p2[[1]]

ggsave(p2[[1]], file="p2.pdf", width=7, height=7)

p3 <- mr_funnel_plot(res_single)

p3[[1]]

ggsave(p3[[1]], file="p3.pdf", width=7, height=7)
