# Supplementary material for: Exploring causality between bone mineral density and frailty: A bidirectional Mendelian randomization study
Source: PLoS One. 2024 Jan 25;19(1):e0296867. doi: 10.1371/journal.pone.0296867 (PMC10810463; doi:10.1371/journal.pone.0296867)
Supplement: Attachment — Submitted filename: Response to Reviewers.docx [file pone.0296867.s003.docx]

Dear Editor and Reviewer,

We sincerely thank the academic editor and the reviewer for their valuable feedback. The insights provided have been instrumental in enhancing the quality of our manuscript. The comments from the academic editor and the reviewer are listed in *italics*, with specific questions numbered for clarity. Our responses are presented in a normal font, and changes or additions made to the manuscript are highlighted in yellow.

Manuscript: PONE-D-23-23648

“Exploring causality between bone mineral density and frailty: a bidirectional Mendelian randomization study”
Reviewer comments:

1. *Line 29: “We performed genome-wide association statistics for BMD” – please correct this, as you did not perform a GWAS.*

Response: We have corrected that at line 29 in revised manuscript. “We utilized summary statistics data for BMD at different skeletal sites”.

2. *Line 32: “different skeletal sites (N=195,723)” – please state the exact sample size for each of the skeletal sites analysed.*

Response: We have revisited the data and made corrections to list the exact sample size for each skeletal site. Changes are in lines 30-33 of the revised manuscript. “including heel BMD (e-BMD, N=40,613), forearm BMD (FA-BMD, N=8,143), femoral neck BMD (FN-BMD, N=32,735), and lumbar spine BMD (LS-BMD, N=28,489), alongside frailty index (FI, N=175,226) data in participants of European ancestry.”

3. *Line 34: “We found that low heel (e) BMD led to an increase in FI” – please rephrase this to say that you found a negative causal estimate between genetically predicted heel BMD and FI. Your study is not a longitudinal study and the use of phrases such as ‘led to increase’ should be avoided.*

Response: Thanks for pointing out this inappropriate expression. We have changed this at lines 37-40 in the revised manuscript. “We observed negative causal estimates between genetically predicted e-BMD (IVW *β* = - 0.020, 95% confidence interval (CI) = - 0.038, - 0.002, *P* = 0.029) and FA-BMD (IVW *β* = -0.035, 95% CI = -0.066, -0.004, *P* = 0.028) with FI.”

4. *Line 34-37: Report either the confidence interval or the p-value, as these two give the same information.*

Response: We have added the β, 95% CI and P values for those two in lines 42-44 of the revised manuscript. “There was no causal effect of FN-BMD (IVW *β* = - 0.024, 95% CI = -0.052, 0.004, *P* = 0.088) and LS-BMD (IVW *β* = - 0.005, 95% CI = -0.034, 0.024, *P* = 0.749) on FI”.

5. *Line 55: Please give a reference for his statement.*

Response: Thanks for pointing out this unsupported statement. We have changed this statement and provided a reference in lines 62-63 of the revised manuscript. “The prevalence of primary osteoporosis increases with age and differs by race/ethnicity [3].”

6. *Line 107: Please replace “pooled data” with “summary statistics”.*

Response: We have replaced “pooled data” with “summary statistics” in line 113 of the revised manuscript.

7. *Line 122-126: Please state the sample size for each of the sites analysed in the manuscript text (as you did in the S1 File) as the 53236 samples does not refer to all of them.*

Response: We have revisited the data and corrected the exact sample sizes for each of the different skeletal sites (lines 131-136). “Summary statistics of FN-BMD (N=32,735), LS-BMD (N=28,489), and FA-BMD (N=8,143) were obtained from the Genetic Factors in Osteoporosis Consortium [19] (GeFOS) GWAS meta-analysis (53,236 participants, European origin). The e-BMD dataset (N=40,613) was obtained from the MRC Integrative Epidemiology Unit (MRC-IEU) consortium and is based on GWAS summary statistics from the UKB.”

We also have listed the sample sizes for the different cohorts of the frailty index (lines 126-127). “UK Biobank (UKB, N=164,610) and TwinGene, Sweden (N=10,616)”.

8. *Line 127-128: This is where you have a problem. “e-BMD dataset were from the UKB GWAS summary statistics, including 142,487 participants of European descent” – you have a substantial overlap between your FI dataset (which is based on a GWAS in UK Biobank) and the heel BMD which is also based on a GWAS in UKB. This leads to biased causal estimates because of sample overlap. Ideally, the exposure and outcome datasets in a two-sample MR setting should not be overlapping. For a continuous outcome, bias due to sample overlap is a linear function of the proportion of overlap between the samples (see https://doi.org/10.1002/gepi.21998). I have checked the GWAS catalog and the ieu open gwas project. There is only one GWAS on frailty index and all of the GWASs on heel BMD are based on UKB. So, I am aware that you will not be able to find a dataset that does not overlap (correct me if I’m wrong), meaning that you need to be aware that your only significant finding in this manuscript could be a false positive.*

Response: We extend our gratitude to the reviewer for highlighting this critical issue. We apologize for our oversight. Upon careful examination, we acknowledge that there is an unavoidable sample overlap in our datasets. The summary statistics for the frailty index are derived from both the UK Biobank (UKB, N=164,610) and TwinGene, Sweden (N=10,616). Unfortunately, we were unable to locate an independent dataset for the TwinGene group. We have estimated the probability of false positives occurring > 50% through the website (https://sb452.shinyapps.io/overlap/). Given this possibility, we have taken a conservative stance regarding the interpretability of this positive result. We have now included a discussion of the sample overlap and its implications in the appropriate sections of the revised manuscript, notably within the data description (lines 139-143), the results (lines 248-250), the discussion (lines 365-373), and the conclusion sections (lines 47-49, 414-416) to ensure full transparency with our readers.

Lines 139-143: “The e-BMD dataset and most of the FI datasets are derived from the UK Biobank, so there will be an overlap of samples [20]. Unfortunately, we were unable to locate an independent dataset for the TwinGene group, and therefore false-positive results may occur (<https://sb452.shinyapps.io/overlap/>).”

Lines 248-250: “Due to the considerable sample overlap between the e-BMD and FI datasets, caution is warranted when considering the suggested negative causal relationship.”

Lines 365-373: “First, The observed potential negative causality between the e-BMD dataset and the FI dataset must be interpreted with caution due to a substantial sample overlap, which could increase the risk of false positive findings. This limitation must be carefully considered when evaluating the results of our analysis. As the availability of GWAS datasets expands, we anticipate conducting future research without such overlap to validate and refine our findings.”

Lines 47-49 and 414-416: “Our study provides support for the hypothesis that low BMD may be a potential causal risk factor for frailty, but further research is needed to confirm this relationship.”, “In conclusion, the findings of this MR study offer suggestive evidence that low bone mineral density (BMD) may be a potential risk factor for frailty”.

9. and 10. *Line 133-135: “First, we utilized the PLINK clumping method within the two-sample MR tool to aggregate SNPs that were significantly associated with the exposure in the GWAS summary data.” – this is not what you did as I can see from the code that you are not using PLINK but used the built-in function extract_instruments from the TwoSampleMR package which returns a set of LD clumped SNPs that are GWAS significant for your exposure. Also, keep in mind that clumping is used for pruning based on LD (second step), and that the first step is just setting a significance threshold to find the significantly associated SNPs.*

*Line 136-140: “To ensure the independence of the selected instrumental variables, we further excluded SNPs that showed linkage disequilibrium (LD) based on European data from the 1,000 Genomes Project as a reference panel (R2 > 0.001 and kb < 10,000) [21].” – again, this was all done by using the built-in extract_instruments function. Please reference the function and the package.*

Response: Thank you for your careful reading of our methods and the observations regarding our SNP selection process. Upon revisiting our analytical approach, we realize that our initial description may have incorrectly suggested the use of PLINK for the clumping procedure. We appreciate your clarification, and we confirm that our code indeed employs the “extract_instruments” function from the TwoSampleMR package to obtain a set of LD clumped SNPs that are GWAS significant for our exposure. Additionally, we acknowledge the importance of differentiating between the setting of the significance threshold to identify SNPs associated with the exposure and the subsequent LD clumping step.

We have revised the manuscript to accurately reflect the methodology we employed and to ensure a clear understanding of the steps taken during our analysis. (lines 146-152 of the revised manuscript) “In the first step of our Mendelian randomization analysis, we applied a significance threshold (*p* < 5 × 10^-8^) to identify genetic instruments that were strongly associated with the exposure in the GWAS summary data. Subsequently, we performed linkage disequilibrium (LD) clumping using the “extract_instruments” function within the TwoSampleMR package, setting an R² threshold of greater than 0.001 (R^2^ > 0.001) and considering a clumping window of 10,000 kilobases (kb).”

11. *Line 140-141: “ This step helps ensure that the instrumental variables used in the analysis are not correlated due to their proximity to the genome.” – what is a proximity to the genome?*

Response: Thank you for seeking clarification on the phrasing used in our description of the instrumental variable (IV) selection process. The term “proximity to the genome” was an imprecise expression that we intended to mean “physical proximity to each other on the genome,” which refers to SNPs that are close to one another on a chromosomal level.

We have revised the statement at lines 152-154 of our manuscript. “This function facilitated the selection of independent SNPs as instruments for our exposure, based on combined GWAS significance and LD structure.”

12. *Line 159: change "signed" to "assigned".*

Response: Thanks for pointing out the spelling error!

We have made the change in line 172 of the revised manuscript. “were assigned to the same alleles”.

13. *Line 163-164: “To detect and correct for pleiotropy, we also performed two analyses” – the MR-Egger intercept is a trivial method to check for horizontal pleiotropy, I just don’t see how can you check for horizontal pleiotropy by using weighted median? Also, neither of these two can correct for horizontal pleiotropy. If you want to correct for uncorrelated horizontal pleiotropy then you can use MR-PRESSO for example, and if you want to correct for correlated horizontal pleiotropy you can use the CAUSE method.*

Response: We appreciate your constructive feedback on our initial approach to pleiotropy in our Mendelian randomization (MR) analysis. In light of your comments, we have now implemented the MR-PRESSO global test in our revised manuscript to specifically assess the presence of horizontal pleiotropy. We recognize the value in using this method to strengthen the validity of our causal inference, and the results from the MR-PRESSO analysis have been incorporated into our findings (Table 3). This has provided an additional layer of scrutiny to our MR analysis and further enhanced the reliability of our conclusions. Through MR-PRESSO analysis, we found that the original e-BMD (ebi-a-GCST006979) dataset had horizontal pleiotropy, so we replaced the e-BMD dataset (ukb-b-11364) and reran the MR analysis.

The revised results from this updated analysis have been integrated into the manuscript, providing a more reliable and robust basis for our conclusions.

Lines 176-178: “To test for horizontal pleiotropy, we also performed two analyses including the MR-PRESSO global test [26] and MR Egger intercept [27].”

Lines 244-247: “Nonetheless, the combined results from the MR Egger intercept test and the MR-PRESSO global test indicated that the Mendelian randomization analysis remained robust, unaffected by any potential horizontal pleiotropy (*P* > 0.05) (**Table 3**).”

Lines 275-278: “In addition, the combined results of the MR-Egger intercept test and the MR-PRESSO global test showed that the MR analysis was not affected by any potential horizontal pleiotropy (*P* > 0.05) (**Table 3**).”

We have also updated the data sources and results sections to reflect these changes.

Line 30: “including heel BMD (e-BMD, N=40,613)”

Lines 134-136: “The e-BMD dataset (N=40613) was obtained from the MRC Integrative Epidemiology Unit (MRC-IEU) consortium and is based on GWAS summary statistics from the UKB.”

14. *Line 178-179: Specify the “problems with multiple testing”.*

Response: Thank you for your request for further specification regarding the problems associated with multiple testing. In our study, we conducted separate MR analyses through four different skeletal sites, treating BMD as the exposure and FI as the outcome. This approach leads to multiple comparisons, which inherently increases the risk of committing type I errors—false positives. To account for this, we applied the Bonferroni correction method by adjusting our significance threshold to a p-value of 0.0125, calculated as 0.05 divided by the number of exposure variables (4). Because of sample overlap in the e-BMD data, we were conservative in our results and therefore used the Bonferroni correction, the most stringent correction for multiple testing.

We have revised the statement at lines 191-195 of our manuscript. “In our study, we performed MR analysis of BMD as exposure and FI as outcome for each of the four different skeletal sites. In view of the multiple tests, the main results were statistically significant at *P* values < 0.0125 (0.05/4) after Bonferroni correction.”

15. *Line 182: Thank you for providing the R code.*

Response: We thank the reviewer for acknowledging our inclusion of the R code in the manuscript. We believe that sharing our code is crucial for the transparency and reproducibility of our work.

*16. Line 192: Where do these 557 SNPs come from? When I extract the instruments for each of the BMD measure sites separately, I get 513 instruments for heel BMD (ebi-a-GCST006979), 21 instruments for femoral neck BMD (ieu-a-980), 3 instruments for forearm BMD ('ieu-a-977') and 24 instruments for lumbar spine BMD (ieu-a-982).*

*You should divide the results section by each of the exposure analysed: 1. Heel BMD, 2. FN BMD, 3. FA BMD and 4. LS BMD, so 4 paragraphs in the Results section for the forward direction MR.*

*Also, you have an error in your code: exp_dat <- extract_instruments(c('ebi-a-GCST006979','ieu-a-980','ieu-a-977','ieu-a-982'))*

*You are extracting all of the exposures together, this then gives you a combined dataset of 561 SNPs from all 4 exposures and in the next step you are using the: out_dat <- extract_outcome_data(snps = exp_dat$SNP, outcomes = 'ebi-a-GCST90020053'), which is extracting SNPs significant and LD-pruned for all 4 exposures together from the outcome dataset. This is incorrect and you should separate the analyses for each of the exposure-outcome combinations, for example:*

*exp_dat <- extract_instruments('ebi-a-GCST006979')*

*and then*

*out_dat <- extract_outcome_data(snps = exp_dat$SNP,*

*outcomes = 'ebi-a-GCST90020053')*

Response: Thank you for the constructive suggestion to divide the Results section to reflect the separate analyses for each BMD exposure site. We agree that such an organizational structure will enable a clearer presentation of the findings and will facilitate an easier comprehension for our readers.

Therefore, we have modified the results section of the manuscript. (lines 207-208) “In our study, we extracted 53 SNPs for e-BMD, 21 SNPs for FN-BMD, 3 SNPs for FA-BMD, and 24 SNPs for LS-BMD, respectively.”

We have also found the error in the R code that you pointed out. The corrected R code can be found in the S1 File (line 570) of the revised manuscript.

16. *Line 193-194: “All of them are associated with FI at genome-wide significance.” – this is not true.*

Response: We thank the reviewer for scrutinizing our manuscript and pointing out this error! We acknowledge that this statement was indeed incorrect and we have corrected this error in the revised manuscript (lines 209-210 and 263-264). “The MR results of this section were based on the SNPs screened under the genome-wide significance threshold (*p* < 5 × 10^-8^)”

17. *Line 204-205: “Lastly, 543 SNPs were taken as IVs for BMD” – BMD is not one exposure, but 4, please correct this.*

Response: Once again, we appreciate your contribution to improving the accuracy of our work.

We have corrected this error in the revised manuscript (lines 221-222). “Lastly, 51 SNPs for e-BMD, 21 SNPs for FN-BMD, 3 SNPs for FA-BMD, and 24 SNPs for LS-BMD were taken as IVs.”

18. *The same remarks apply to the presentation of results in the reverse direction. Please, divide it by the 4 outcomes, so 4 paragraphs in the Results section for the reverse direction MR.*

Response: We have corrected this error in the revised manuscript (lines 261-262). “Similarly, we extracted 15 SNPs for e-BMD, 14 SNPs for FN-BMD, 14 SNPs for FA-BMD, and 14 SNPs for LS-BMD, respectively.”

19. *Also, there is no need to perform the leave-one-out analysis if the primary finding is not significant.*

Response: We appreciate your guidance regarding the use of leave-one-out analysis in the context of our findings. We concur with your assessment that if the primary finding of the MR analysis is not statistically significant, it becomes less important to perform a leave-one-out to examine the effect of individual single nucleotide polymorphisms (SNPs) on the overall outcome. In light of your comment, we have reevaluated the necessity of reporting the leave-one-out analysis within our manuscript, given that our primary findings did not reach statistical significance. Following this reevaluation, we have removed mention of the leave-one-out analysis from the results. (lines 186-188 and 279-283)

20. *Supplementary files: Try to combine them into fewer files. For example, file S1 is just 1 table, it can be combined with file S2 and file S5.*

*Both files S3 and S4 should be formatted in a way that is easy to read, right now it is impossible to read them as they are in the csv format. Use the library(WriteXLS) to write these data into an xlsx file and combine the two files into one xlsx file with 2 sheets.*

Response: Thank you for your valuable suggestions on reorganizing the supplementary files to enhance readability and accessibility.

We have consolidated Supplementary Table S1 with Supplementary Files S2 and S5 into a single file (S1 File in line 570).

We have reformatted the data previously contained within Supplementary Files S3 and S4 for ease of readability. We have converted these CSV files into more reader-friendly Excel (xlsx) format. These two files have now been combined into one Excel workbook, consisting of two separate sheets, each corresponding to the original files S3 and S4 (S2 File in line 571).

All supplementary files have been carefully renamed to reflect these changes and to ensure consistency across the entire manuscript. The references to these files within the text have also been updated accordingly.

Journal requirements (academic editor):

1. *Please ensure that your manuscript meets PLOS ONE's style requirements, including those for file naming.*

Response: Thank you for your guidance and for pointing out the style requirements of PLOS ONE. I appreciate the opportunity to ensure that our manuscript aligns with the journal's standards. I have carefully reviewed the style and file naming requirements listed in the PLOS ONE guidelines. I have revised the manuscript and supporting documentation accordingly to ensure full compliance.

Line 12: (YL)

Line 256: Table 1.

Line 297: Table 2.

Line 301: Table 3.

Lines 570-571: S1 File S2 File

2. *We noticed you have some minor occurrence of overlapping text with the following previous publication(s), which needs to be addressed:*

*https://doi.org/10.1016/j.joca.2021.12.001*

*In your revision ensure you cite all your sources (including your own works), and quote or rephrase any duplicated text outside the methods section.*

Response: Thank you for bringing this issue to our attention. Please be assured that we take the matter of proper citation and avoidance of text duplication very seriously. To address this concern, we have thoroughly reviewed our manuscript to identify and rectify any instances of overlapping text. In the revised manuscript, you will find all the changes highlighted (lines 116-120, 364-365, 371-373, 390-392, and 396-400) which were made to specifically address the issue of overlapping text.

Lines 116-120: “In the bidirectional Mendelian randomization (MR) study design, dashed arrows represent the assumption that genetic variants are neither associated with confounders nor directly influencing the outcome, and such paths should ideally be absent in an MR analysis. The blue and red paths illustrate the bidirectional nature of two-sample MR.”

Lines 364-365: “This study has several limitations that should be considered when interpreting the results.”

Lines 371-373: “Second, our study is potentially limited by the relatively small number of SNPs that were used as genetic instruments.”

Lines 390-392: “To ensure that the insights gleaned from Mendelian randomization are universally relevant, further studies incorporating diverse ethnic groups are essential.”

Lines 396-400: “Despite the limitations, the present study also boasts several advantages that reinforce the validity of our findings. First, we utilized a comprehensive approach by including genetic variants associated with BMD not only from a single skeletal site but from multiple anatomical locations, capturing a more systemic genetic influence on BMD.”

Once again, we express our gratitude for the time and attention dedicated by the editor and reviewer to our work. We believe that these revisions have significantly improved our manuscript and look forward to any further suggestions you may have.

Kind regards,

Corresponding author: Yi Lu
